# In adenosine A_2B_ knockouts acute treatment with inorganic nitrate improves glucose disposal, oxidative stress, and AMPK signaling in the liver

**DOI:** 10.3389/fphys.2015.00222

**Published:** 2015-08-07

**Authors:** Maria Peleli, Michael Hezel, Christa Zollbrecht, A. Erik G. Persson, Jon O. Lundberg, Eddie Weitzberg, Bertil B. Fredholm, Mattias Carlström

**Affiliations:** ^1^Department of Physiology and Pharmacology, Karolinska InstitutetStockholm, Sweden; ^2^Department of Medical Cell Biology, Uppsala UniversityStockholm, Sweden

**Keywords:** insulin resistance, metabolic syndrome, NADPH oxidase, nitric oxide, nitrite, superoxide, obesity, type 2 diabetes

## Abstract

**Rationale:** Accumulating studies suggest that nitric oxide (NO) deficiency and oxidative stress are central pathological mechanisms in type 2 diabetes (T2D). Recent findings demonstrate therapeutic effects by boosting the nitrate-nitrite-NO pathway, which is an alternative pathway for NO formation. This study aimed at investigating the acute effects of inorganic nitrate on glucose and insulin signaling in adenosine A_2B_ receptor knockout mice (A^−/−^_2B_), a genetic mouse model of impaired metabolic regulation.

**Methods:** Acute effects of nitrate treatment were investigated in aged wild-type (WT) and A^−/−^_2B_ mice. One hour after injection with nitrate (0.1 mmol/kg, i.p.) or placebo, metabolic regulation was evaluated by intraperitoneal glucose and insulin tolerance tests. NADPH oxidase-mediated superoxide production and AMPK phosphorylation were measured in livers obtained from non-treated or glucose-treated mice, with or without prior nitrate injection. Plasma was used to determine insulin resistance (HOMA-IR) and NO signaling.

**Results:** A^−/−^_2B_ displayed increased body weight, reduced glucose clearance, and attenuated overall insulin responses compared with age-matched WT mice. Nitrate treatment increased circulating levels of nitrate, nitrite and cGMP in the A^−/−^_2B_, and improved glucose clearance. In WT mice, however, nitrate treatment did not influence glucose clearance. HOMA-IR increased following glucose injection in the A^−/−^_2B_, but remained at basal levels in mice pretreated with nitrate. NADPH oxidase activity in livers from A^−/−^_2B_, but not WT mice, was reduced by nitrate treatment. Livers from A^−/−^_2B_ displayed reduced AMPK phosphorylation compared with WT mice, and this was increased by nitrate treatment. Finally, injection with the anti-diabetic agent metformin induced similar therapeutic effects in the A^−/−^_2B_ as observed with nitrate.

**Conclusion:** The A^−/−^_2B_ mouse is a genetic mouse model of metabolic syndrome. Acute treatment with nitrate improved the metabolic profile in it, at least partly via reduction in oxidative stress and improved AMPK signaling in the liver.

## Introduction

Metabolic syndrome, which worsens during aging and obesity, is a cluster of biochemical and physiological abnormalities that increase the risk of developing cardiovascular disease and type 2 diabetes (T2D) (Eckel et al., 2005; Carlström, 2011). Reduced nitric oxide (NO) production from endothelial nitric oxide synthase (eNOS) and augmented oxidative stress are proposed to be central events in metabolic syndrome (Litvinova et al., 2015). In the past decade, an alternative pathway for NO formation has been described where inorganic nitrate is serially reduced to nitrite and then NO and other bioactive nitrogen oxides (Lundberg et al., 2008, 2009, 2011). We have shown that several features of metabolic syndrome present in aged eNOS-deficient mice can be reversed by dietary supplementation with inorganic nitrate (Carlström et al., 2010). A recent study showed that chronic nitrite supplementation through increased phosphorylation of the skeletal muscle AMP activated kinase (AMPK) improved some metabolic syndrome components in a model of obesity (Singamsetty et al., 2015). Moreover, chronic treatment with nitrate attenuates oxidative stress and high blood pressure in models of renal and cardiovascular disease (Carlström et al., 2011a; Gao et al., 2015).

Adenosine is another important regulator of metabolism, and signaling via its different receptor subtypes, A_1_, A_2A_, A_2B_, and A_3_, has also gained a lot of interest (Chen et al., 2013). In a recent publication we demonstrated that abrogation of adenosine A_1_ signaling improves metabolic regulation in aged mice by modulating nicotinamide adenine dinucleotide phosphate (NADPH) oxidase activity and immune responses (Yang et al., 2015a). Besides the A_1_ receptor, signaling via both A_2A_ and A_2B_ receptors play important roles in modulating glucose homeostasis and fat mass (Johnston-Cox et al., 2012; Gnad et al., 2014). Another recent study suggested gene deletion of adenosine A_2B_ receptor as a suitable model for metabolic syndrome (Csóka et al., 2014). The authors showed that A_2B_ knockout mice (A^−/−^_2B_), fed a regular chow, displayed increased body weight and fat mass, impaired glucose and insulin homeostasis, together with dysregulated insulin, adipokine, triglyceride, and cholesterol metabolism compared with wild-type (WT) control mice. Food consumption was similar between genotypes, but daily walking time was reduced in the A^−/−^_2B_ mice. Moreover, Johnston-Cox et al. reported that a high fat diet (HFD) aggravated the abnormal metabolic phenotype in A^−/−^_2B_, whereas Csoka and colleagues did not observe this.

The current study aimed at investigating the acute effects of inorganic nitrate treatment on metabolic functions in aged A_2B_ receptor knockout mice (A^−/−^_2B_). Considering previous findings about nitrate- or nitrite-mediated modulation of both NADPH oxidase (Montenegro et al., 2011; Carlström et al., 2011a; Gao et al., 2015; Yang et al., 2015b) and AMPK (Kamga Pride et al., 2014; Singamsetty et al., 2015), we hypothesized that nitrate could improve abnormal metabolic functions during aging and increased fat mass by increasing AMPK activation and moderating oxidative stress. We observed improved metabolic regulation in the A^−/−^_2B_ mice after nitrate treatment and this was indeed associated with decreased NADPH oxidase-derived superoxide production in the liver, possibly mediated via restored AMPK activation.

## Materials and methods

### Animals

**Figure 1:**
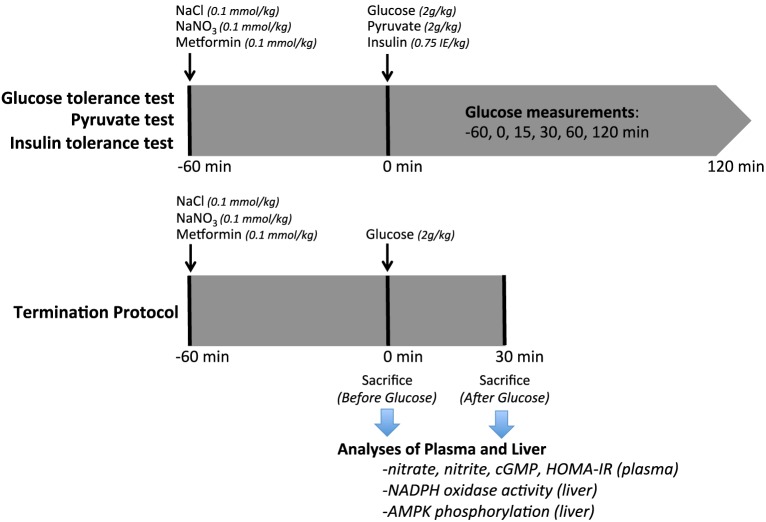
**Experimental Protocol**. Diagram of the experimental procedures on aged WT or A_2B_KO mice undergoing intraperitoneal glucose (IPGTT), pyruvate or insulin (IPITT) tolerance tests. In the termination series, plasma and liver were collected at 0 or 30 min. NaCl, sodium chloride; NaNO_3_, sodium nitrate; IPGTT, intraperitoneal glucose tolerance test; IPITT, intraperitoneal insulin tolerance test.

This study was approved by the Institutional Animal Care and Use Committee (IACUC) in Stockholm, and performed according to the National Institutes of Health guidelines for the conduct of experiments in animals. Experiments were conducted on aged (12–16 months) adenosine A_2B_ receptor gene-deleted and WT mice from heterozygous breeding pairs. A^−/−^_2B_ mice (a gift from professor M. Sitkovsky at Northwestern University, Boston, Mass) were backcrossed 11 times to a C57BL/6J background at Northwestern University. Both sexes were used, with equal distribution for all experimental series. Mice were housed in temperature-controlled rooms with 12 h light/dark cycles and received a standard rodent chow (4% fat, R34, Lactamin AB, Kimstad, Sweden) and tap water *ad libitum*. An overview of the experimental protocol is shown in Figure 1.

### Intraperitoneal glucose, insulin, and pyruvate tolerance tests

Glucose tolerance tests (IPGTT) were performed following 6 h of fasting, as described previously (Yang et al., 2015a). Inorganic nitrate (NaNO_3_; 0.1 mmol/kg body weight) or placebo (NaCl, 0.1 mmol/kg body weight) was administered intraperitoneally 60 min prior to the tolerance tests. In a human (70 kg) this dose of nitrate corresponds to around 450 mg; an amount found in a single serving of a nitrate rich vegetable such as spinach, beetroot, or lettuce (Weitzberg and Lundberg, 2013). A bolus of D-glucose or pyruvate was injected (2 g/kg body weight; 30% in saline) and tail blood was sampled at 0, 15, 30, 60, and 120 min. Plasma glucose was determined using a portable glucose meter (FreeStyle Lite, Abbot Diabetes Care Inc, CA). In a cohort of A^−/−^_2B_ mice we also investigated the effects on glucose disposal with the anti-diabetic drug metformin. Metformin (0.1 mmol/kg body weight) or placebo (NaCl, 0.1 mmol/kg body weight) was administered intraperitoneally 60 min prior to the IPGTT. Homeostasis model assessment-estimated insulin resistance (HOMA-IR) was calculated at baseline, at 60 min after injection with nitrate or placebo, and again 30 min after injection with glucose.

In order to investigate the acute effects of nitrate in a model with more pronounced obesity, IPGTT were performed as described above in WT mice given a HFD (34.9% fat, D12492, Research Diets Inc., New Brunswick, NJ) for 14 months (Supplementary Material).

Intraperitoneal insulin tolerance tests (IPITT) were performed similarly to IPGTT without fasting. A bolus of insulin (0.75 IE/kg body weight; Novorapid 100 IE/ml, Novo Nordisk A/S, Denmark) was injected (0.2 IE/ml in saline) and blood samples were obtained for plasma glucose measurements.

### Plasma analysis

Insulin content was measured using ELISAs purchased from Mercodia (Uppsala, Sweden). Plasma samples containing IBMX (10 μM) were analyzed for cGMP using ELISA method (EIA system; GE Healthcare). All kits were run according to manufacturers' instructions. Nitrite and nitrate were analyzed by HPLC (ENO-20) and autosampler (840, EiCom, Kyoto, Japan), as described previously (Carlström et al., 2010; Hezel et al., 2015). The plasma samples were extracted using methanol (1:2) and then centrifuged for 10 min (4°C; 10.000 g), separated by reverse phase/ion exchange chromatography followed by nitrate reduction to nitrite by cadmium and reduced copper. The nitrite was then derivatized using Griess reagent to form diazo compounds and analyzed by detection at 540 nm.

### NADPH oxidase activity

NADPH oxidase-mediated superoxide formation was detected by lucigenin-dependent chemiluminescence assay (Carlstrom et al., 2013; Yang et al., 2015a). Livers were separately homogenized and used for subsequent activity measurement.

### Western blotting of AMPK

Livers obtained from mice under (1) basal condition, (2) after pretreatment with placebo, nitrate or metformin, and (3) after stimulation with glucose were weighed and homogenized using 0.5 mm zirconium oxide beads (Bullet Blender™, Next Advance, Inc., Stockholm, Sweden) in 2.5 volumes of lysis buffer containing 10 mM Tris-HCl (pH 8), 150 mM NaCl, 5 mM EDTA, 60 mM N-octyl glucoside, 1% Triton X-100, protease, and phosphatase inhibitor cocktails (Sigma-Aldrich, Stockholm, Sweden). After centrifugation and protein quantification of the soluble fraction (Protein Assay Dye Reagent Concentrate; Bio-Rad Laboratories, Solna, Sweden), equal amounts of protein were separated by SDS-PAGE followed by transfer to a PVDF membrane (Bio-Rad). The membranes were blocked with 5% nonfat dry milk in Tween-containing TBS, incubated with specific primary antibody for phosphorylated AMPK (Thr172; Cell signaling/BioNordika, Stockholm, Sweden) and anti-rabbit secondary antibody (horseradish peroxidase-conjugated goat antibody to rabbit IgG, Santa Cruz, Heidelberg, Germany). To detect total AMPK, Restore™ PLUS Western Blot Stripping Buffer (Thermo Scientific™, Göteborg, Sweden) was applied followed by blocking and re-probing the membranes with primary antibody for AMPK (Cell Signaling/BioNordika) and anti-rabbit secondary antibody. Protein bands were visualized using Clarity Western ECL Substrate (Bio-Rad), intensities were quantified using densitometry (Image Lab 5.2.1 software, Bio-Rad) and results are reported as relative optical density of the specific proteins.

### Statistical analysis

Values are presented as means ± SEM. Single comparisons between normally distributed parameters were tested for significance using the Student's paired or unpaired *t*-test as appropriate. For multiple group comparisons, One-Way ANOVA followed by Bonferroni's *post-hoc* test was used to allow for more than one comparison with the same variable. Statistical significance was defined as *p* < 0.05.

## Results

### Animal characteristics

Body weight was significantly higher in aged (12–16 months) A^−/−^_2B_ (36.5 ± 0.8 g; *n* = 42) compared with age-matched WT mice (32.5 ± 0.9 g; *n* = 40), and plasma glucose levels in non-fasting mice were also higher in the A^−/−^_2B_ mice (9.5 ± 0.4 vs. 7.6 ± 0.2 mmol/L). WT mice fed with HFD for 14 months were more obese (body weight; 60.6 ± 3.3 g; *n* = 10) compared with the aged-matched mice on a regular chow (*P* < 0.05).

### Glucose tolerance tests

**Figure 2:**
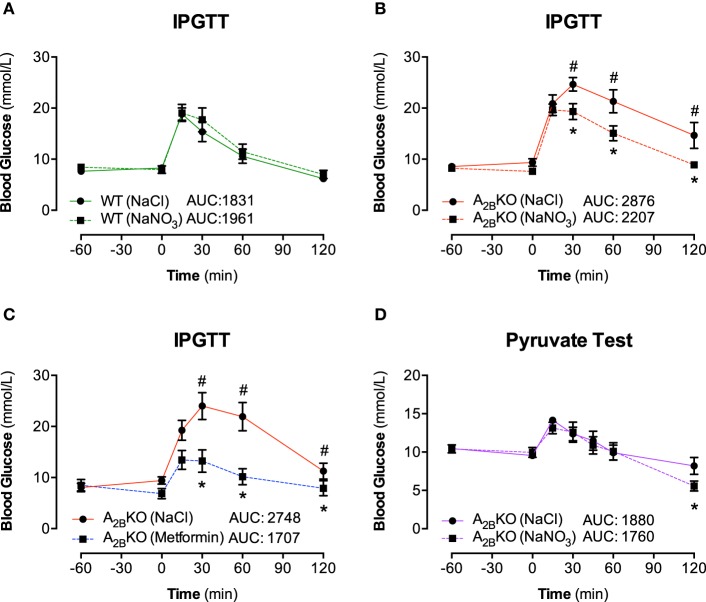
**IPGTT and Pyruvate test**. The effect of inorganic nitrate on glucose tolerance was determined by measuring plasma glucose levels in WT mice **(A)** and A_2B_KO mice **(B)** after placebo (NaCl) or nitrate injection (NaNO_3_). Glucose levels after injection of metformin were determined in A_2B_KO mice to investigate the effect of the anti-diabetic drug in mice with metabolic syndrome **(C)**. The impaired glucose tolerance in A_2B_KO mice compared to WT could be significantly improved both with NaNO_3_ and with metformin. Finally, to assess any potential effect of nitrate on gluconeogenesis, a pyruvate tolerance test was performed **(D)**. The total AUC (mmol/L/min) for the 0–120 min period was calculated. Values are mean ± SEM, *n* = 10–16/group. ^*^p < 0.05 vs. A_2B_KO (NaCl); ^#^*p* < 0.05 vs. WT.

To investigate the ability of acute inorganic nitrate treatment to modulate the metabolic phenotype in aged A^−/−^_2B_ mice we performed glucose tolerance tests in aged A^−/−^_2B_ and WT mice 1 h after nitrate injection (NaNO_3_; 0.1 mmol/kg body weight). Fasting blood glucose levels were similar in A^−/−^_2B_ and WT mice and nitrate treatment had no influence on glucose clearance in WT mice (Figure 2A). Interestingly, the impaired glucose tolerance in A^−/−^_2B_ mice compared to WT was significantly improved after acute nitrate treatment (Figure 2B). Administration of the anti-diabetic drug metformin to the A^−/−^_2B_ mice resulted in an even more pronounced increase in glucose tolerance (Figure 2C). Similar to that observed in A^−/−^_2B_ mice, acute treatment with nitrate also improved glucose disposal (AUC 1493 ± 91 vs. 1779 ± 118; *n* = 10) in HFD-treated mice (Supplementary Material).

### Pyruvate tolerance test

We probed whether nitrate influences gluconeogenesis in A^−/−^_2B_ mice, which could contribute to the production and clearance of glucose. To this end, the administration of the gluconeogenic substrate precursor pyruvate showed that there was no difference in glucose production in mice with nitrate pretreatment. Hence, nitrate had no significant impact on the gluconeogenesis pathway. However, upon glucose production (after 60 min), the clearance rates of glucose were again significantly faster in nitrate treated A^−/−^_2B_ mice compared to placebo group (Figure 2D; 120 min), confirming a promotion in glucose clearance upon acute treatment with inorganic nitrate.

### Insulin tolerance tests and HOMA-IR

**Figure 3:**
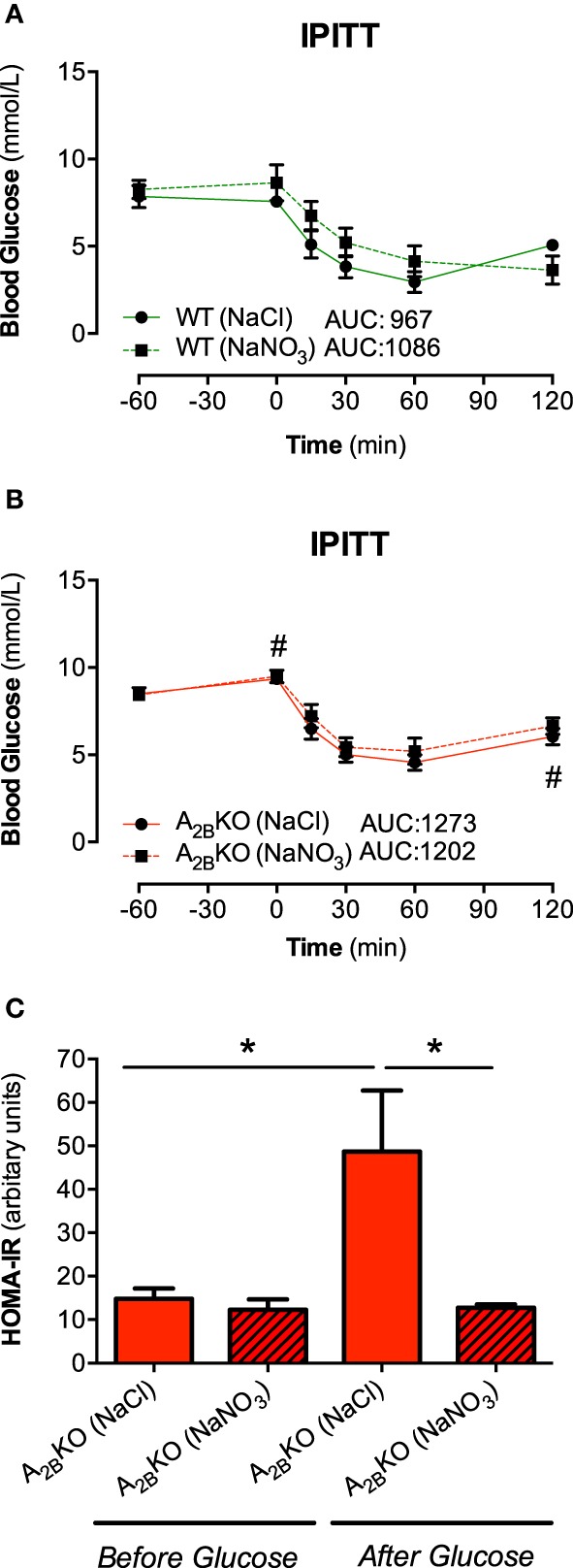
**IPITT and HOMA-IR**. Plasma glucose levels in response to insulin injection were not influenced by nitrate treatment in WT **(A)** and A_2B_KO mice **(B)**. HOMA-IR, a measure of insulin resistance, increased following glucose injection in A_2B_KO mice, which was prevented by prior nitrate treatment **(C)**. Values are mean ± SEM, *n* = 10–16/group. ^#^*p* < 0.05 vs. WT; ^*^p < 0.05 among the indicated groups.

Insulin sensitivity after insulin injection (IPITT) was lower in A^−/−^_2B_ mice (Figure 3B) compared to WT (Figure 3A) resulting in higher AUC. Glucose clearance did not differ between placebo or nitrate treated animals of both genotypes. In addition, insulin resistance (expressed as HOMA-IR) during fasting condition was similar in both genotypes but increased following glucose injection in the A^−/−^_2B_ mice (Figure 3C). This could be prevented by prior injection with nitrate.

### Nitrate, nitrite, and cGMP in plasma

**Figure 4:**
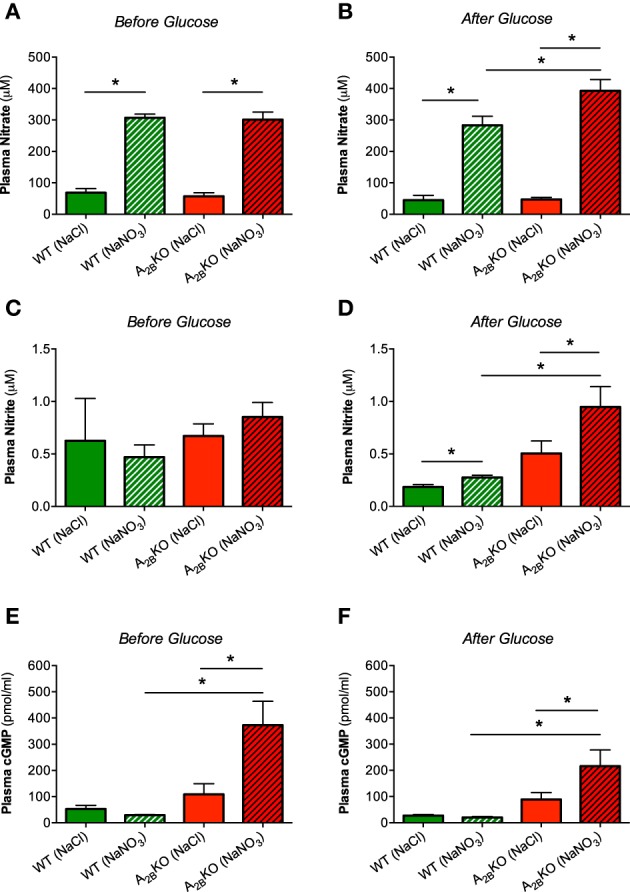
**Nitrate, nitrite and cGMP in plasma**. Treatment with nitrate significantly increased plasma levels of nitrate in both genotypes before **(A)** and after glucose injection **(B)**. Plasma nitrite levels were significantly increased only in nitrate treated A_2B_KO mice after glucose **(C, D)**. In A_2B_KO mice, nitrate supplementation caused a significant increase of plasma cGMP levels both before **(E)** and after glucose injection **(F)**. Values are mean ± SEM, *n* = 6–14/group. ^*^p < 0.05 among the indicated groups.

Nitrate treatment increased plasma nitrate levels in both WT and A^−/−^_2B_ mice as expected (Figure 4A) and this increase was even higher in A^−/−^_2B_ mice compared to WT after glucose injection (Figure 4B). Plasma nitrite levels were not different between groups during fasting (Figure 4C) but after glucose injection, A^−/−^_2B_ mice treated with nitrate showed significantly higher nitrite levels compared to WT and also A^−/−^_2B_ placebo group (Figure 4D). The second messenger cGMP, a central downstream NO signaling target, was not influenced by nitrate or glucose in WT mice (Figures 4E,F). However, in A^−/−^_2B_ mice treatment with nitrate resulted in a significant increase in plasma cGMP levels compared to the placebo group and WT mice.

### NADPH oxidase activity in the liver

**Figure 5:**
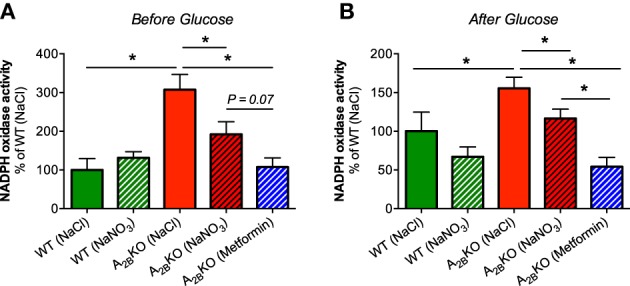
**NADPH oxidase activity in the liver**. NADPH oxidase-derived superoxide formation was measured with lucigenin-dependent chemiluminescence signal in liver homogenates derived from A_2B_KO and WT mice before **(A)** and after glucose injection **(B)**. A significantly increased NADPH oxidase activity was observed in A_2B_KO compared to WT, which could be diminished by prior injection of nitrate or metformin. Values are mean ± SEM, *n* = 6–14/group. ^*^p < 0.05 among the indicated groups.

NADPH oxidase-derived superoxide production in liver homogenates from A^−/−^_2B_ mice was significantly higher compared to WT mice (Figure 5A). Interestingly, nitrate treatment as well as injection of metformin significantly reduced superoxide production whereas nitrate had no effect on NADPH oxidase activity in WT mice. The same beneficial effects of nitrate and metformin were observed after glucose injection (Figure 5B).

### AMPK regulation in the liver

**Figure 6:**
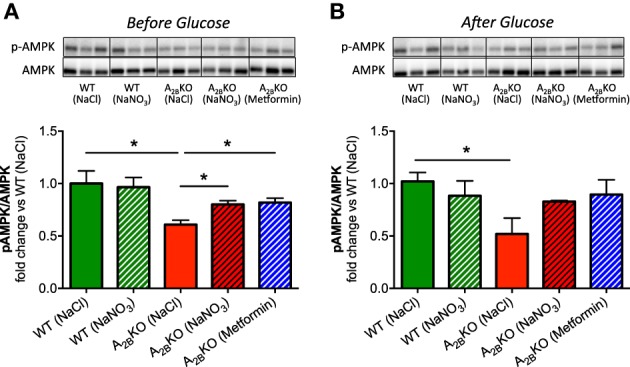
**AMPK regulation in the liver**. Western blot was used to determine AMPK expression and phosphorylation in liver. Before **(A)** and after glucose injection **(B)**, A_2B_KO mice showed significantly lower AMPK activation compared to WT. This could be improved by prior injection of nitrate or metformin. Three representative samples per group obtained from different gels are shown together with densitometric quantification, presented as ratio p-AMPK/AMPK. Values are mean ± SEM, *n* = 4/group. ^*^p < 0.05 among the indicated groups.

Expression and phosphorylation levels of AMPK were assessed in liver tissue derived from all animal groups. The ratio of phosphorylated AMPK to total AMPK, as a measure of AMPK activation, was significantly lower in A^−/−^_2B_ mice compared to WT (Figures 6A,B). This could be partially rescued by treatment with nitrate or metformin. As was the case with the NADPH oxidase activity, AMPK activation could also be improved with nitrate or metformin after glucose injection.

## Discussion

### Aged A^−/−^_2B_ mice present characteristics of the metabolic syndrome

We confirmed previous studies (Johnston-Cox et al., 2012; Csóka et al., 2014) showing that aged A^−/−^_2B_ mice present several features of the metabolic syndrome; they are more obese than WT mice, display hyperglycemia and poor glucose clearance.

### Impaired liver AMPK activation could contribute to the metabolic dysregulation in aged A^−/−^_2B_ mice

The mechanisms leading to the development of this impaired metabolic regulation in the A^−/−^_2B_ mice are still being investigated and there are many unanswered questions. Major contributing factors are elevated hepatic inflammation and IRS-2 expression (Johnston-Cox et al., 2012) and augmented classical macrophage activation in the adipose tissue (Csóka et al., 2014). In this study we focused mainly on the liver since it is an organ of high importance in glucose metabolism and T2D development (Bechmann et al., 2012) and has not been extensively investigated in A^−/−^_2B_ mice. AMPK is an important intracellular energy sensor and one of the key players in maintaining liver glucose homeostasis (Viana et al., 2006; Wang et al., 2012), which is often downregulated under hyperglycemic conditions (Kraegen et al., 2006). The ability to activate liver AMPK is a major reason why the anti-diabetic drugs metformin and 5-amino-4-imidazolecarboxamide riboside (AICAR) were developed and used as treatments (Towler and Hardie, 2007). In our study we show for the first time that aged A^−/−^_2B_ mice present lower phosphorylation levels of liver AMPK compared to normoglycemic WT mice of similar age. In agreement with that, the AMPK activator metformin elevated liver AMPK phosphorylation levels together with a remarkable improvement of glucose clearance. Thus, one may speculate that impaired AMPK activation is one of the factors contributing to metabolic dysfunction in this animal model.

### Elevated liver NADPH oxidase activity could contribute to the metabolic dysregulated phenotype of the aged A^−/−^_2B_ mice

Another key regulator of liver glucose uptake and metabolism is the enzyme family of NADPH oxidases. It is well known that elevated levels of NADPH oxidase-derived superoxide in the liver diminish glucose uptake and contribute to the development of hyperglycemia (Guichard et al., 2008). To our knowledge, this is the first study showing that A^−/−^_2B_ mice present higher levels of liver NADPH oxidase activity compared to WT, both before and after glucose treatment. Therefore, the investigation of pharmacological interventions to target liver NADPH oxidases may be of great interest for the improvement of the metabolic phenotype when the A_2B_ receptors are ablated.

### AMPK and NADPH oxidases are closely linked

Several studies have indicated that pharmacological activation of AMPK can reduce NADPH oxidase activity and expression in various target organs and cells like liver (Adachi and Brenner, 2008), cardiomyocytes (Balteau et al., 2014), podocytes (Piwkowska et al., 2010), and human umbilical vein endothelial cells (Ceolotto et al., 2007). One mechanism leading to reduced production reactive oxygen species (ROS) might be via upregulated mRNA expression of the antioxidant enzymes SOD2 and catalase, as it was seen in activated hepatic stellate cells treated with AMPK activators AICAR or metformin (Adachi and Brenner, 2008). Another mechanism of how activated AMPK can inhibit NADPH oxidase activity was shown in activated human neutrophils where AMPK activation with AICAR prevented phosphorylation and membrane translocation of the cytosolic NADPH oxidase subunit p47phox, which are both crucial to the enzyme activation (Alba et al., 2004). However, it is still unknown if this inhibition of NADPH oxidases and ROS production by p-AMPK can lead to improved glucose clearance. Speaking in favor of this concept, activation of AMPK by inorganic nitrate or metformin in our study was clearly associated with both reduced NADPH oxidase activity and improved glucose tolerance in A^−/−^_2B_ mice. Functional AMPK may therefore be important as an early warning system for oxidative stress to trigger compensatory antioxidant effects, which in turn improve glucose uptake. Future studies are needed to investigate if inorganic nitrate exerts its beneficial effect via upregulation of antioxidant enzymes, prevention of p47phox phosphorylation and translocation or another mechanism.

### The nitrate-nitrite-NO pathway is upregulated in the aged A^−/−^_2B_ mice

We and other groups have previously shown that long-term treatment with inorganic nitrate or nitrite improves glucose clearance, insulin sensitivity, and reduces visceral fat levels during aging and obesity (Carlström et al., 2010; Hezel et al., 2015; Singamsetty et al., 2015). However, the underlying mechanisms of how inorganic nitrate and nitrite mediate their beneficial effects remain to be elucidated. In the current study we investigated if acute treatment with inorganic nitrate can exert similar effects as observed with long-term supplementation. Interestingly, nitrate treatment significantly increased the plasma levels of nitrite and cGMP only in A^−/−^_2B_ mice, but not in the WT, and the increase in plasma nitrate levels was higher in A^−/−^_2B_ mice compared with WT after glucose load. Since the baseline levels of nitrate, nitrite and cGMP were similar between WT and A^−/−^_2B_ it seems that the nitrate-nitrite-NO pathway is sensitized in the absence of A_2B_ receptors. Activation of the A_2_ receptors has been linked with increased NOS activation, and therefore it is likely that the NOS function is compromised in the A_2B_ knockouts. There are publications showing that A_2_ receptor signaling is associated with higher NO production in the renal microvasculature (Carlström et al., 2011b) and in the liver during ischemia/reperfusion injury (Peralta et al., 1999). Moreover, A_2_ receptor activation, especially of the type 2B, leads to higher NO production in coronary artery endothelial cells (Olanrewaju and Mustafa, 2000) and enhances vasorelaxation in mouse aorta (Ansari et al., 2007). Another mechanism potentiating the action of nitrate in the A^−/−^_2B_ mice but not in WT might be via the higher superoxide levels. Several studies in redox biology suggest that stimulating NO production or antioxidant systems are more potent when there are higher levels of ROS and in particular superoxide (Wink et al., 2001; Silva et al., 2012; Araujo and Wilcox, 2014). Since activation of A_2B_ receptors can facilitate NO production and mice lacking these receptors already present higher levels of liver superoxide one could speculate that this oxidative stress leads to a more prominent and faster activation of the alternative nitrate-nitrite-NO pathway.

### The nitrate-nitrite-NO pathway in T2D: clinical and experimental data

In recent bibliography there are several experimental *in vivo* and *in vitro* studies showing favorable effects of inorganic nitrate and nitrite in T2D (Bahadoran et al., 2015). The proposed mechanisms involve compensation for disturbed eNOS-derived NO generation (Carlström et al., 2010), improved antioxidant capacity (Khalifi et al., 2015), and increased pancreatic islet blood flow and insulin secretion (Nyström et al., 2012). Moreover, nitrate/nitrite-mediated NO production may also improve insulin resistance and glucose uptake by activation of glucose transporter 4 (GLUT4) (Jiang et al., 2014; Ohtake et al., 2015). Apart from the experimental reports some clinical studies have been conducted. So far there is evidence that inorganic nitrate or nitrite could have beneficial effects on overweight or slight obese patients or in T2D despite no clear correlation between T2D and plasma or urinary levels of nitrate and nitrite (Bahadoran et al., 2015). Joris et al. showed that supplementation with beetroot juice, which is high in inorganic nitrate, improved postpranial endothelial function in slight overweight or obese men (Joris and Mensink, 2013). In patients with T2D, a single dose of inorganic nitrate was suggested to lower basal plasma glucose and improve oral glucose insulin sensitivity index, however no improvement in glucose tolerance was observed (Cermak et al., 2015). In another small study in patients with T2D, Gilchrist and colleagues did not observe improvement in endothelial function or insulin sensitivity (Gilchrist et al., 2013), but their findings suggested that dietary nitrate could improve cognitive function in diabetic patients (Gilchrist et al., 2014). Taken together, despite several studies reporting beneficial properties with inorganic nitrate and nitrite, the data from small size clinical studies are still contradictory and clearly show the need for a carefully conducted large-scale, long-term follow up trial in patients.

In summary, the present study demonstrates an important influence of acute inorganic nitrate treatment in modulating metabolic functions. In aged A_2B_ receptor knockout mice, characterized by metabolic syndrome, inorganic nitrate improved their glucose clearance and this was associated with increased AMPK activation and reduced NADPH oxidase activity in the liver. Similar favorable effects of acute nitrate on glucose disposal was also observed in HFD-treated obese WT mice. Intriguingly, the dose of nitrate was similar to what is found in a single serving of a green leafy vegetable; the predominant dietary source of nitrate. These findings suggest that the beneficial effects of inorganic nitrate act not only long-term but also acutely and future studies should be aimed at determining the therapeutic value of dietary nitrate supplementation in patients with metabolic disease.

### Conflict of interest statement

Jon O. Lundberg and Eddie Weitzberg are co-inventors on patent applications related to the therapeutic use of inorganic nitrate. The authors declare that the research was conducted in the absence of any commercial or financial relationships that could be construed as a potential conflict of interest.

## References

[B1] Adachi M. Brenner D. A. (2008). High molecular weight adiponectin inhibits proliferation of hepatic stellate cells via activation of adenosine monophosphate-activated protein kinase. Hepatology 47, 677–685. 10.1002/hep.21991 18220291

[B2] Alba G. El Bekay R. Alvarez-Maqueda M. Chacón P. Vega A. Monteseirín J.. (2004). Stimulators of AMP-activated protein kinase inhibit the respiratory burst in human neutrophils. FEBS Lett. 573, 219–225. 10.1016/j.febslet.2004.07.077 15328001

[B3] Ansari H. R. Nadeem A. Talukder M. A. Sakhalkar S. Mustafa S. J. (2007). Evidence for the involvement of nitric oxide in A2B receptor-mediated vasorelaxation of mouse aorta. Am. J. Physiol. Heart Circ. Physiol. 292, H719–H725. 10.1152/ajpheart.00593.2006 16920807

[B4] Araujo M. Wilcox C. S. (2014). Oxidative stress in hypertension: role of the kidney. Antioxid. Redox Signal. 20, 74–101. 10.1089/ars.2013.5259 23472618 PMC3880923

[B5] Bahadoran Z. Ghasemi A. Mirmiran P. Azizi F. Hadaegh F. (2015). Beneficial effects of inorganic nitrate/nitrite in type 2 diabetes and its complications. Nutr. Metab. (Lond). 12:16. 10.1186/s12986-015-0013-6 25991919 PMC4436104

[B6] Balteau M. Van Steenbergen A. Timmermans A. D. Dessy C. Behets-Wydemans G. Tajeddine N.. (2014). AMPK activation by glucagon-like peptide-1 prevents NADPH oxidase activation induced by hyperglycemia in adult cardiomyocytes. Am. J. Physiol. Heart Circ. Physiol. 307, H1120–H1133. 10.1152/ajpheart.00210.2014 25128166

[B7] Bechmann L. P. Hannivoort R. A. Gerken G. Hotamisligil G. S. Trauner M. Canbay A. (2012). The interaction of hepatic lipid and glucose metabolism in liver diseases. J. Hepatol. 56, 952–964. 10.1016/j.jhep.2011.08.025 22173168

[B8] Carlström M. (2011). Seasonal variation in metabolic syndrome components: how much do they influence the diagnosis of metabolic syndrome? Curr. Cardiovasc. Risk Rep. 5, 29–37. 10.1007/s12170-010-0139-z

[B9] Carlstrom M. Brown R. D. Yang T. Hezel M. Larsson E. Scheffer P. G.. (2013). L-arginine or tempol supplementation improves renal and cardiovascular function in rats with reduced renal mass and chronic high salt intake. Acta Physiol. (Oxf.) 207, 732–741. 10.1111/apha.12079 23387940

[B10] Carlström M. Larsen F. J. Nyström T. Hezel M. Borniquel S. Weitzberg E.. (2010). Dietary inorganic nitrate reverses features of metabolic syndrome in endothelial nitric oxide synthase-deficient mice. Proc. Natl. Acad. Sci. U.S.A. 107, 17716–17720. 10.1073/pnas.1008872107 20876122 PMC2955084

[B11] Carlström M. Persson A. E. Larsson E. Hezel M. Scheffer P. G. Teerlink T.. (2011a). Dietary nitrate attenuates oxidative stress, prevents cardiac and renal injuries, and reduces blood pressure in salt-induced hypertension. Cardiovasc. Res. 89, 574–585. 10.1093/cvr/cvq366 21097806

[B12] Carlström M. Wilcox C. S. Welch W. J. (2011b). Adenosine A2A receptor activation attenuates tubuloglomerular feedback responses by stimulation of endothelial nitric oxide synthase. Am. J. Physiol. Renal Physiol. 300, F457–F464. 10.1152/ajprenal.00567.2010 21106859 PMC3044007

[B13] Ceolotto G. Gallo A. Papparella I. Franco L. Murphy E. Iori E.. (2007). Rosiglitazone reduces glucose-induced oxidative stress mediated by NAD(P)H oxidase via AMPK-dependent mechanism. Arterioscler. Thromb. Vasc. Biol. 27, 2627–2633. 10.1161/ATVBAHA.107.155762 17916771

[B14] Cermak N. M. Hansen D. Kouw I. W. van Dijk J. W. Blackwell J. R. Jones A. M.. (2015). A single dose of sodium nitrate does not improve oral glucose tolerance in patients with type 2 diabetes mellitus. Nutr. Res. [Epub ahead of print]. 10.1016/j.nutres.2015.05.017 26092495

[B15] Chen J. F. Eltzschig H. K. Fredholm B. B. (2013). Adenosine receptors as drug targets–what are the challenges? Nat. Rev. Drug Discov. 12, 265–286. 10.1038/nrd3955 23535933 PMC3930074

[B16] Csóka B. Koscsó B. Töro G. Kókai E. Virág L. Németh Z. H.. (2014). A2B adenosine receptors prevent insulin resistance by inhibiting adipose tissue inflammation via maintaining alternative macrophage activation. Diabetes 63, 850–866. 10.2337/db13-0573 24194503 PMC3931402

[B17] Eckel R. H. Grundy S. M. Zimmet P. Z. (2005). The metabolic syndrome. Lancet 365, 1415–1428. 10.1016/S0140-6736(05)66378-7 15836891

[B18] Gao X. Yang T. Liu M. Peleli M. Zollbrecht C. Weitzberg E.. (2015). NADPH oxidase in the renal microvasculature is a primary target for blood pressure-lowering effects by inorganic nitrate and nitrite. Hypertension 65, 161–170. 10.1161/HYPERTENSIONAHA.114.04222 25312440

[B19] Gilchrist M. Winyard P. G. Aizawa K. Anning C. Shore A. Benjamin N. (2013). Effect of dietary nitrate on blood pressure, endothelial function, and insulin sensitivity in type 2 diabetes. Free Radic. Biol. Med. 60, 89–97. 10.1016/j.freeradbiomed.2013.01.024 23395779

[B20] Gilchrist M. Winyard P. G. Fulford J. Anning C. Shore A. C. Benjamin N. (2014). Dietary nitrate supplementation improves reaction time in type 2 diabetes: development and application of a novel nitrate-depleted beetroot juice placebo. Nitric Oxide 40, 67–74. 10.1016/j.niox.2014.05.003 24858657

[B21] Gnad T. Scheibler S. von Kügelgen I. Scheele C. Kilic A. Glöde A.. (2014). Adenosine activates brown adipose tissue and recruits beige adipocytes via A2A receptors. Nature 516, 395–399. 10.1038/nature13816 25317558

[B22] Guichard C. Moreau R. Pessayre D. Epperson T. K. Krause K. H. (2008). NOX family NADPH oxidases in liver and in pancreatic islets: a role in the metabolic syndrome and diabetes? Biochem. Soc. Trans. 36, 920–929. 10.1042/BST0360920 18793162

[B23] Hezel M. P. Liu M. Schiffer T. A. Larsen F. J. Checa A. Wheelock C. E.. (2015). Effects of long-term dietary nitrate supplementation in mice. Redox Biol. 5, 234–242. 10.1016/j.redox.2015.05.004 26068891 PMC4475696

[B24] Jiang H. Torregrossa A. C. Potts A. Pierini D. Aranke M. Garg H. K.. (2014). Dietary nitrite improves insulin signaling through GLUT4 translocation. Free Radic. Biol. Med. 67, 51–57. 10.1016/j.freeradbiomed.2013.10.809 24157451

[B25] Johnston-Cox H. Koupenova M. Yang D. Corkey B. Gokce N. Farb M. G.. (2012). The A2b adenosine receptor modulates glucose homeostasis and obesity. PLoS ONE 7:e40584. 10.1371/journal.pone.0040584 22848385 PMC3405065

[B26] Joris P. J. Mensink R. P. (2013). Beetroot juice improves in overweight and slightly obese men postprandial endothelial function after consumption of a mixed meal. Atherosclerosis 231, 78–83. 10.1016/j.atherosclerosis.2013.09.001 24125415

[B27] Kamga Pride C. Mo L. Quesnelle K. Dagda R. K. Murillo D. Geary L.. (2014). Nitrite activates protein kinase A in normoxia to mediate mitochondrial fusion and tolerance to ischaemia/reperfusion. Cardiovasc. Res. 101, 57–68. 10.1093/cvr/cvt224 24081164 PMC3868348

[B28] Khalifi S. Rahimipour A. Jeddi S. Ghanbari M. Kazerouni F. Ghasemi A. (2015). Dietary nitrate improves glucose tolerance and lipid profile in an animal model of hyperglycemia. Nitric Oxide 44, 24–30. 10.1016/j.niox.2014.11.011 25461274

[B29] Kraegen E. W. Saha A. K. Preston E. Wilks D. Hoy A. J. Cooney G. J.. (2006). Increased malonyl-CoA and diacylglycerol content and reduced AMPK activity accompany insulin resistance induced by glucose infusion in muscle and liver of rats. Am. J. Physiol. Endocrinol. Metab. 290, E471–E479. 10.1152/ajpendo.00316.2005 16234268

[B30] Litvinova L. Atochin D. N. Fattakhov N. Vasilenko M. Zatolokin P. Kirienkova E. (2015). Nitric oxide and mitochondria in metabolic syndrome. Front. Physiol. 6:20. 10.3389/fphys.2015.00020 25741283 PMC4330700

[B31] Lundberg J. O. Carlström M. Larsen F. J. Weitzberg E. (2011). Roles of dietary inorganic nitrate in cardiovascular health and disease. Cardiovasc. Res. 89, 525–532. 10.1093/cvr/cvq325 20937740

[B32] Lundberg J. O. Gladwin M. T. Ahluwalia A. Benjamin N. Bryan N. S. Butler A.. (2009). Nitrate and nitrite in biology, nutrition and therapeutics. Nat. Chem. Biol. 5, 865–869. 10.1038/nchembio.260 19915529 PMC4038383

[B33] Lundberg J. O. Weitzberg E. Gladwin M. T. (2008). The nitrate-nitrite-nitric oxide pathway in physiology and therapeutics. Nat. Rev. Drug Discov. 7, 156–167. 10.1038/nrd2466 18167491

[B34] Montenegro M. F. Amaral J. H. Pinheiro L. C. Sakamoto E. K. Ferreira G. C. Reis R. I.. (2011). Sodium nitrite downregulates vascular NADPH oxidase and exerts antihypertensive effects in hypertension. Free Radic. Biol. Med. 51, 144–152. 10.1016/j.freeradbiomed.2011.04.005 21530643

[B35] Nyström T. Ortsater H. Huang Z. Zhang F. Larsen F. J. Weitzberg E.. (2012). Inorganic nitrite stimulates pancreatic islet blood flow and insulin secretion. Free Radic. Biol. Med. 53, 1017–1023. 10.1016/j.freeradbiomed.2012.06.031 22750508

[B36] Ohtake K. Nakano G. Ehara N. Sonoda K. Ito J. Uchida H.. (2015). Dietary nitrite supplementation improves insulin resistance in type 2 diabetic KKA(y) mice. Nitric Oxide 44, 31–38. 10.1016/j.niox.2014.11.009 25461271

[B37] Olanrewaju H. A. Mustafa S. J. (2000). Adenosine A(2A) and A(2B) receptors mediated nitric oxide production in coronary artery endothelial cells. Gen. Pharmacol. 35, 171–177. 10.1016/S0306-3623(01)00107-0 11744240

[B38] Peralta C. Hotter G. Closa D. Prats N. Xaus C. Gelpí E.. (1999). The protective role of adenosine in inducing nitric oxide synthesis in rat liver ischemia preconditioning is mediated by activation of adenosine A2 receptors. Hepatology 29, 126–132. 10.1002/hep.510290104 9862858

[B39] Piwkowska A. Rogacka D. Jankowski M. Dominiczak M. H. Stepinski J. K. Angielski S. (2010). Metformin induces suppression of NAD(P)H oxidase activity in podocytes. Biochem. Biophys. Res. Commun. 393, 268–273. 10.1016/j.bbrc.2010.01.119 20123087

[B40] Silva B. R. Pernomian L. Bendhack L. M. (2012). Contribution of oxidative stress to endothelial dysfunction in hypertension. Front. Physiol. 3:441. 10.3389/fphys.2012.00441 23227009 PMC3514688

[B41] Singamsetty S. Watanabe Y. Guo L. Corey C. Wang Y. Tejero J.. (2015). Inorganic nitrite improves components of the metabolic syndrome independent of weight change in a murine model of obesity and insulin resistance. J. Physiol. 593, 3135–3145. 10.1113/JP270386 25952686 PMC4532532

[B42] Towler M. C. Hardie D. G. (2007). AMP-activated protein kinase in metabolic control and insulin signaling. Circ. Res. 100, 328–341. 10.1161/01.RES.0000256090.42690.05 17307971

[B43] Viana A. Y. Sakoda H. Anai M. Fujishiro M. Ono H. Kushiyama A.. (2006). Role of hepatic AMPK activation in glucose metabolism and dexamethasone-induced regulation of AMPK expression. Diabetes Res. Clin. Pract. 73, 135–142. 10.1016/j.diabres.2005.12.011 16503364

[B44] Wang S. Song P. Zou M. H. (2012). AMP-activated protein kinase, stress responses and cardiovascular diseases. Clin. Sci. 122, 555–573. 10.1042/CS20110625 22390198 PMC3367961

[B45] Weitzberg E. Lundberg J. O. (2013). Novel aspects of dietary nitrate and human health. Annu. Rev. Nutr. 33, 129–159. 10.1146/annurev-nutr-071812-161159 23642194

[B46] Wink D. A. Miranda K. M. Espey M. G. Pluta R. M. Hewett S. J. Colton C.. (2001). Mechanisms of the antioxidant effects of nitric oxide. Antioxid. Redox Signal. 3, 203–213. 10.1089/152308601300185179 11396476

[B47] Yang T. Gao X. Sandberg M. Zollbrecht C. Zhang X. M. Hezel M.. (2015a). Abrogation of adenosine A1 receptor signalling improves metabolic regulation in mice by modulating oxidative stress and inflammatory responses. Diabetologia 58, 1610–1620. 10.1007/s00125-015-3570-3 25835725

[B48] Yang T. Peleli M. Zollbrecht C. Giulietti A. Terrando N. Lundberg J. O.. (2015b). Inorganic nitrite attenuates NADPH oxidase-derived superoxide generation in activated macrophages via a nitric oxide-dependent mechanism. Free Radic. Biol. Med. 83, 159–166. 10.1016/j.freeradbiomed.2015.02.016 25724690

