# Supplementary material for: In adenosine A2B knockouts acute treatment with inorganic nitrate improves glucose disposal, oxidative stress, and AMPK signaling in the liver
Source: Front Physiol. 2015 Aug 7;6:222. doi: 10.3389/fphys.2015.00222 (PMC4528163; doi:10.3389/fphys.2015.00222)
Supplement: Supplementary file 1 [file DataSheet1.PDF]

Running title: *Inorganic nitrate improves metabolic function*

Online Supplement

Glucose tolerance tests (IPGTT) were performed following 6 hours of fasting. Inorganic nitrate (NaNO3; 0.1 mmol/kg body weight) or placebo (NaCl, 0.1 mmol/kg body weight) was administered intraperitoneally 60 min prior to the tolerance tests. In a human (70 kg) this dose of nitrate corresponds to around 450 mg; an amount found in a single serving of a nitrate rich vegetable such as spinach, beetroot or lettuce (Weitzberg and Lundberg, 2013). A bolus of D-glucose or pyruvate was injected (2g/kg body weight; 30% in saline) and tail blood was sampled at 0, 15, 30, 60, and 120 min. Plasma glucose was determined using a portable glucose meter (FreeStyle Lite, Abbot Diabetes Care Inc, CA).

In order to investigate the acute effects of nitrate (NO_3) in a model with more pronounced obesity, glucose tolerance tests were performed in wild-type (WT) mice given a high-fat diet (HFD; 34.9% fat, D12492, Research Diets Inc, New Brunswick, NJ) for 14 months.
Glucose tolerance tests (IPGTT) were performed following 6 hours of fasting. Inorganic nitrate (NaNO3; 0.1 mmol/kg body weight) or placebo (NaCl, 0.1 mmol/kg body weight) was administered intraperitoneally 60 min prior to the tolerance tests. A bolus of D-glucose was injected (2g/kg body weight; 30% in saline) and tail blood was sampled at 0, 15, 30, 60, and 120 min. Plasma glucose was determined using a portable glucose meter (FreeStyle Lite, Abbot Diabetes Care Inc, CA). The experiments were conducted in a paired crossover manner with one-week washout.

Our results show that administration of nitrate, prior to glucose challenge, significantly improved glucose tolerance (**Fig. S1**).

Figure S1.

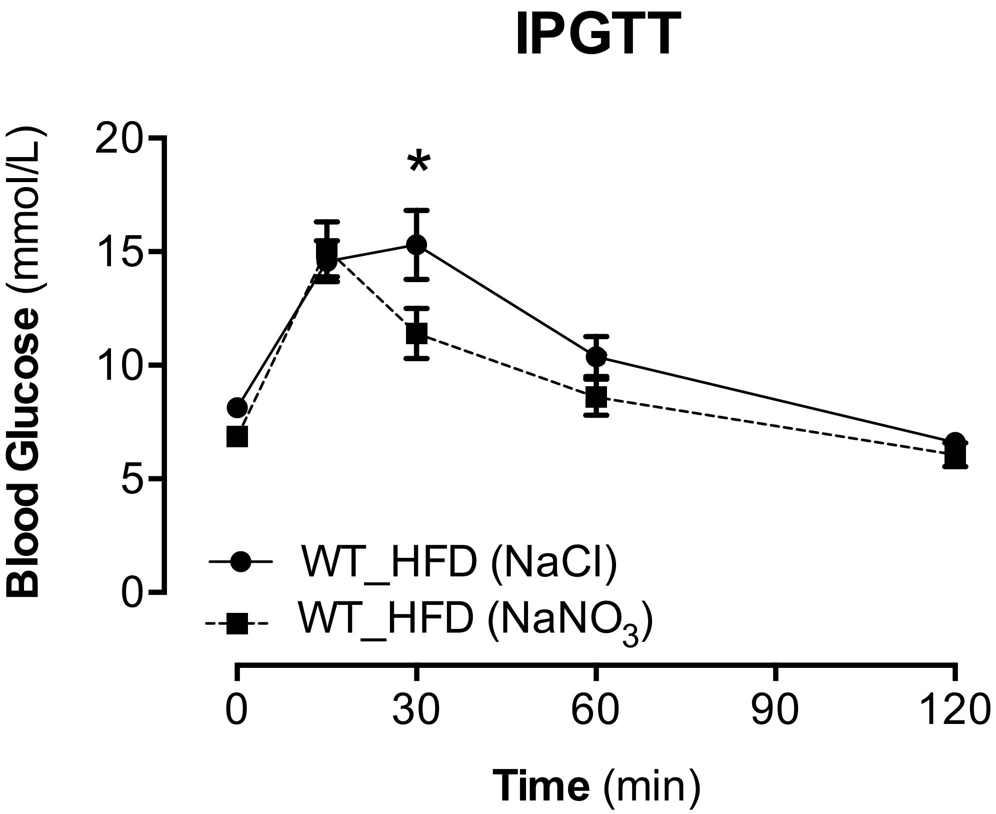

Intraperitoneal Glucose Tolerance Tests (IPGTT)

The effect of inorganic nitrate on glucose tolerance was determined by measuring plasma glucose levels in WT mice fed with high fat diet (HFD). The blood glucose responses were studied after placebo (NaCl) or nitrate injection ($NaNO_3$). The glucose tolerance was significantly improved with $NaNO_3$ as compared with placebo treatment (AUC 1493±91 vs 1779±118; $p<0.05$) in HFD-treated mice Values are mean±SEM, n=10/group. *p<0.05 vs same time-point.
